# The Genetics of Alzheimer’s Disease in the Chinese Population

**DOI:** 10.3390/ijms21072381

**Published:** 2020-03-30

**Authors:** Chen-Ling Gan, Tao Zhang, Tae Ho Lee

**Affiliations:** Fujian Key Laboratory for Translational Research in Cancer and Neurodegenerative Diseases, Institute for Translational Medicine, School of Basic Medical Sciences, Fujian Medical University, Fuzhou 350122, China; ganchenling@mail.fjmu.edu.cn (C.-L.G.); taozh@fjmu.edu.cn (T.Z.)

**Keywords:** Alzheimer’s disease, amyloid precursor protein, presenilin, apolipoprotein E, Chinese population

## Abstract

Alzheimer’s disease (AD) is a neurodegenerative disease characterized by progressive cognitive dysfunction and behavioral impairment. In China, the number of AD patients is growing rapidly, which poses a considerable burden on society and families. In recent years, through the advancement of genome-wide association studies, second-generation gene sequencing technology, and their application in AD genetic research, more genetic loci associated with the risk for AD have been discovered, including *KCNJ15*, *TREM2,* and *GCH1*, which provides new ideas for the etiology and treatment of AD. This review summarizes three early-onset AD causative genes (*APP*, *PSEN1*, and *PSEN2*) and some late-onset AD susceptibility genes and their mutation sites newly discovered in China, and briefly introduces the potential mechanisms of these genetic susceptibilities in the pathogenesis of AD, which would help in understanding the genetic mechanisms underlying this devastating disease.

## 1. Introduction

Based on data from the World Alzheimer Report, 2018, there were approximately 50 million dementia cases around the world in 2018, with the number projected to reach 152 million by 2050 [1]. Although dementia has been the fastest growing epidemic in developed countries, with a prevalence of 1 in 10 persons in the population older than 65 years and over 50% in the population over 85 years old [2], most of the increase in global dementia cases will appear in developing countries over the next several decades, especially in rapidly developing Asian countries, such as India and China. It is estimated that approximately 60% of dementia patients live in Asian regions, mainly in low- and middle-income countries [3]. China and India are among the top ten countries with the highest cases diagnosed with dementia. The national census in 2010 reported that people older than 60 years old account for 13.26% of the total Chinese population. The prevalence of age-related diseases and dementia is rising rapidly across the country [4,5]. According to Wang et al., the pooled prevalence rate of dementia in mainland China was 4.9% (95% confidence interval: 4.3–5.4%) from 1985 to 2018, and it is speculated that the number of people affected by dementia will reach 16.93 million, 24.25 million, 31.98 million, and 35.98 million in 2020, 2030, 2040, and 2050, respectively [6]. Alzheimer’s disease (AD), the most common form of dementia, is a major public health problem throughout the world [7]. There were approximately 5.69 million people affected by AD in China in 2010, based on a systematic review by Chan et al. [4]. Since AD usually has a long disease course and lacks effective treatments, the disease has caused tremendous economic burdens to both caregivers and society. Jia et al. carried out a cost-of-illness study on AD patients in China [8]. According to the research, China has a high proportion of the total worldwide costs of dementia (17.52% in 2015) [8]. The annual estimate per patient in China is also higher than the average cost worldwide [8]. The increasing prevalence of AD and its subsequent socio-economic burdens prompt the necessity of identifying the etiology and developing therapeutics for AD, and making it a priority of the health care system. In this review, we provide a brief description of the general genetic basis of different stages of AD worldwide, and summarize newly found mutations in the main risk genes in the Chinese population.

## 2. Genetics of AD

AD is a degenerative disorder characterized by progressive cognitive dysfunction and behavioral impairment [9,10]. The main pathological hallmarks of AD are the formation of senile plaques from amyloid beta (Aβ) peptides and hyperphosphorylated tau protein in the form of neurofibrillary tangles [10]. Currently, it is generally believed that AD is caused by complex interactions between a multitude of genetic, and epigenetic modifications, and environmental factors. Although the strongest known risk factor for AD is advanced age, some individuals may develop AD-type dementia at a younger age due to pathological factors. Based on the age of onset and genetic predisposition, AD can be divided into two subtypes: familial/early onset (EOAD) and sporadic/late onset (LOAD) [11,12,13]. EOAD occurs from 30 to 60 or 65 years old and accounts for 1% to 6% of all AD cases. Three genes have been implicated in EOAD: the amyloid precursor protein (*APP*) gene on chromosome 21q, the presenilin-1 (*PSEN1*) gene on chromosome 14q, and the presenilin-2 (*PSEN2*) gene on chromosome 1q [14]. Mutations in all these familial AD-associated genes influence amyloid formation, accumulation, and/or deposition [14,15,16,17]. Although data from a Swedish twin study demonstrated that LOAD might have a heritability as high as 79% [18], common gene variants from genome-wide association studies (GWAS) can only explain less than 50% of the phenotypic variations in LOAD cases [19], suggesting that genetic and environmental factors play a pivotal role in the onset, development, and prognosis of the disease [20]. The ε4 allele of the apolipoprotein E (*APOE*) gene has been generally recognized as the strongest risk factor for the pathogenesis of LOAD in several populations [21,22], although it is still not clear how *APOE* ε4 induces AD. The application of GWAS in the study of disease-associated genes in the human genome enables the discovery of a number of interesting genetic loci that might play a role in the development and progression of AD [23,24]. The next generation sequencing (NGS) also facilitates the identification of susceptible genes involved in AD [25,26]. However, additional studies are needed to identify other causative genes that might influence AD risk to fully elucidate the etiology of this devastating disease.

### 2.1. EOAD Susceptibility Genes

EOAD accounts for a small percentage of all AD cases, and commonly presents autosomal dominant patterns of inheritance within families. Currently, there are three recognized risk genes responsible for EOAD. Patients with autosomal inheritance diagnosed with dementia under the age of 60 years are usually caused by genetic mutations in these three genes. To date, over 300 mutations have been reported for *APP*, *PSEN1*, and *PSEN2* in different populations according to the repository of variants in genes linked to AD [17].

#### 2.1.1. APP

**Figure 1:**
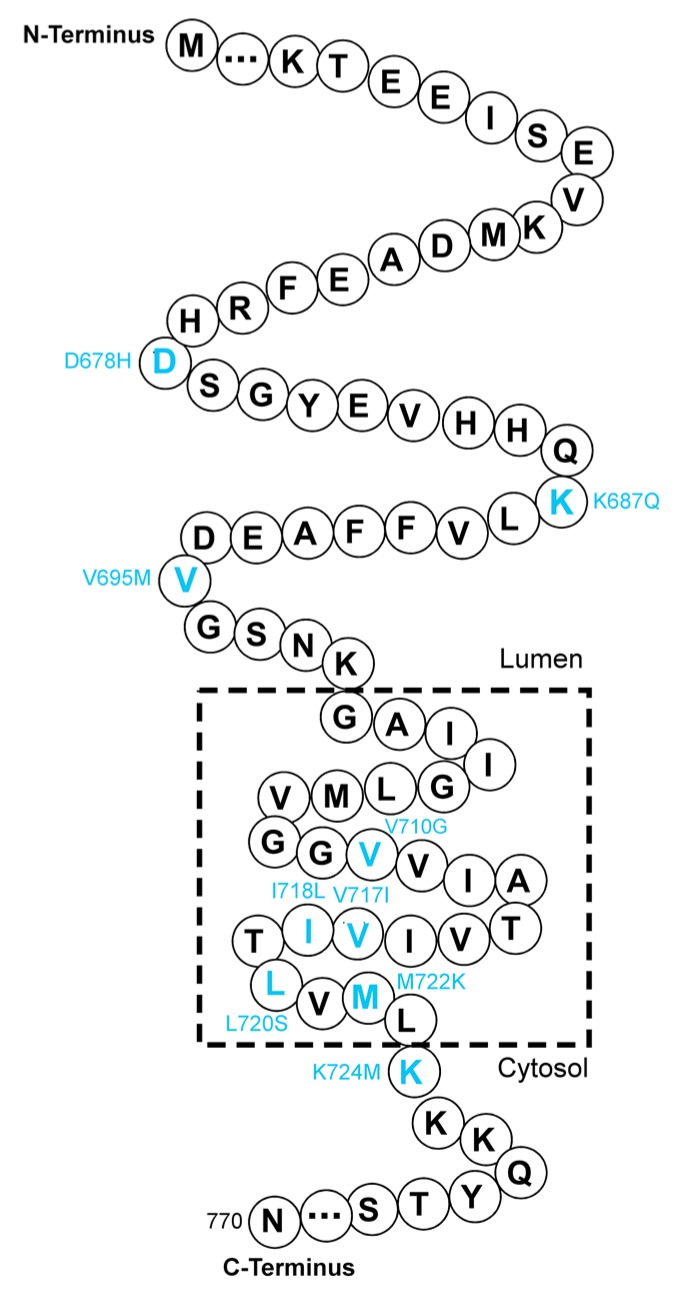
A part of the amino acid sequence of APP770. The reported distribution of mutations around the cleavage sites of APP in the Chinese population is labeled in cyan at the corresponding sites.

The *APP* gene (OMIM 104760), located on chromosome 21q21, encodes APP with three isoforms: APP751, APP770 (Figure 1), and APP695 [27]. APP695 is the major isoform found in the vertebrate brain [28].

The proteolytic processing of APP is mediated by a series of secretases, including α-, β-, and γ-secretases [27]. The amyloidogenic pathway and non-amyloidogenic pathway are the main cleavage pathways of APP in neurons [29]. In the amyloidogenic pathway, APP is successively cleaved by β-, and γ-secretases and Aβ proteins are the major products [27]. Excessive neurotoxic Aβ proteins gradually accumulate in the brain and form amyloid fibrils, which are recognized as a key component of amyloid deposits in the brain parenchyma [30]. In fact, missense mutations in the APP gene were first reported to cause early-onset familial Alzheimer’s disease (EOFAD) [31,32,33,34,35]. Today, more than 50 mutations in *APP* have been found to cause AD, and most of the pathogenic mutations are clustered in exons 16 and 17 of *APP* (around cleavage sites for α-, β-, and γ-secretases, especially γ-secretases), such as p.K687N [36] and p.M722K (Table 1) [37]. Three APP polymorphisms at codons 710, 718, and 720 with single nucleotide substitutions (corresponding to V710G, I718L and L720S) were found first in Taiwanese patients associated with Chinese/Taiwanese patients with AD in 2009 [38]. Jiang et al. carried out whole-exome screening to identify the gene mutations of *APP*, *PSEN1,* and *PSEN2* in a small group of Chinese FAD patients [39]. In addition to the reported *APP* gene mutations (p.V717I), they discovered two novel mutations in APP (p.D244G and p.K687Q) [39]. Moreover, the p.K687Q mutation in APP is a likely pathogenic variant in AD, according to the standards of the American College of Medical Genetics and Genomics (ACMG) [39], while the other mutation (p.D244G) and two mutations (p.T297M and p.D332G), which were previously not associated with AD, remain uncertain with respect to their significance in the pathogenesis of AD [39]. In a similar study by Gao et al., the p.V695M mutation in APP has been found in a patient with amnestic symptoms, but the pathogenicity of this mutation needs further clarification [40]. Peng et al. first reported a Chinese family with autosomal dominant EOAD caused by a heterozygous *APP* gene mutation (p.K724M) [41]. The proband was diagnosed with progressive memory decline and typical executive dysfunction at the age of 45 years. Magnetic resonance imaging further showed global brain atrophy in the brain of the proband [41]. The mutation has been shown to increase the ratio of Aβ42 to Aβ40 in vitro compared with the wild type *APP* control [41]. The involvement of this mutation in Aβ pathology may therefore contribute to the risk of EOFAD in the Chinese population [41]. The first cohort report of the screen of the causative genes of Chinese patients diagnosed with EOFAD was performed by Jiao et al., who reported that a recognized pathogenic mutation of APP (p.V717I) has been detected in two unrelated families with different phenotypes [42]. This finding supports the experimental evidence from Muratore et al., showing that the APP V717I mutation alters the cleavage site of γ-secretase and promotes the generation of both Aβ42 and Aβ38 [43]. This mutation may also exacerbate tau pathology in inducible pluripotent stem cell lines from AD patients [43]. The D678H mutation in APP was identified in Taiwan in 2014 [44]. The middle-aged patient displayed a combined phenotype of dementia and cerebral microvasculopathy, indicating the presence of amyloid deposition both in the brain parenchyma and cerebral vessels [44]. Another novel missense mutation in the *APP* gene, named M722K, has been found in a Chinese EOFAD pedigree [37]. The phenotype of patients with this mutation may be related to their *APOE* genotypes. Using cell models, Wang et al. demonstrated that the M722K mutation also elevates the ratio of Aβ42 to Aβ40 and increases tau phosphorylation at the S202/204 sites [37]. The M722K mutation of APP has been further confirmed in the Chinese Familial Alzheimer’s Disease Network (CFAN) cohort study [45]. It is evident that most of the mutations in APP in familial AD occur around the cleavage site of γ-secretase (Figure 1 and Table 1), which may suggest the significance of these mutations in modulating the generation of Aβ in the brain.

#### 2.1.2. PSEN1

**Figure 2:**
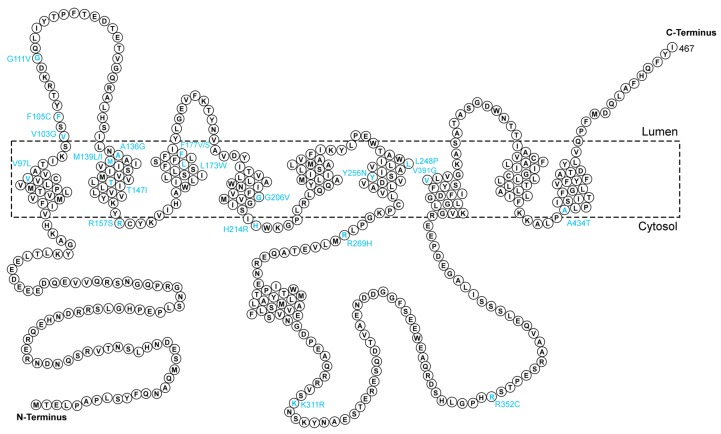
Amino acid sequence of presenilin 1 (PSEN1). The reported distribution of mutations in the Chinese population is labeled in cyan at the corresponding sites.

*PSEN1* is located on chromosome 14q24 [67]. The PSEN1 protein (Figure 2) is the catalytic subunit of γ-secretases, through which APP can be cleaved into Aβ [68]. To date, over 300 penetrant mutations have been reported throughout the world in patients with EOAD (age of onset < 65 years), and the majority of pathogenic *PSEN1* mutations in exons 5, 6, 7, and 8 account for up to 70% of all recorded mutations in EOAD cases. *PSEN1* mutations can affect the function of γ-secretases in neurons, leading to altered Aβ generation and changing the ratio of Aβ42 to Aβ40 [67]. Early studies on the mutations of exon 4/5 of *PSEN1* in AD in China were conducted by using polymerase chain reaction-single strand conformation polymorphism (PCR-SSCP) [52,69]. Since the research was mostly published in domestic journals, and it is difficult to obtain international attention, there is no in-depth follow-up study internationally or domestically. A novel missense mutation (p.V97L), which occurs in the conserved domain of PSEN1, was identified in a Chinese pedigree of FAD in 2005 (Table 1) [49]. This gene mutation only presents in Chinese FAD patients and is likely to induce the development and rapid progression of AD. Further studies indicated the pathological significance of the p.V97L mutations of PSEN1, showing that the mutation increases Aβ42 levels in SH-SY5Y neuroblastoma cells and impedes the transport regulation and intracellular Ca^2+^ homeostasis under endoplasmic reticulum (ER) stress [48,70]. In 2007, the PSEN1 A136G mutation was reported as a genetic risk factor for AD in a Chinese-based study [51]. Several Chinese population-based EOFAD-related *PSEN1* gene mutations were identified by different groups [39,42,50,53,55,61,71,72]. For example, Jiang et al. identified two PSEN1 mutations (p.R352C and p.M233L) in two separate EOFAD families in China [61]. Both mutations are predicted to be strongly associated with disease phenotypes. Interestingly, patients with the p.R352C mutation developed disease symptoms very early and progressed very rapidly, while the other mutation manifested a relatively slow disease course [61]. The p.V391G mutation in PSEN1 was found to be responsible for EOAD with extrapyramidal symptoms according to a study on a very early-onset AD proband in China [62]. Li and colleagues performed Sanger sequencing on *PSEN1* in three Chinese EOAD families and discovered two novel mutations (p.Y256N and p.H214R) in AD families and a de novo mutation (p.G206V) in a patient with very early-onset sporadic AD [57]. The three mutations might contribute to the pathogenesis of EOAD [57,61,62]. A study on an FAD patient cohort in China was conducted by Gao et al. recently, which showed that *PSEN1* mutations account for the largest proportion of FAD patients, and three novel mutations in PSEN1, namely p.M139L, p.V103G, and p.F177V, were identified in the study [40]. Structural analyses indicated that these mutations might induce structural alterations in γ-secretases due to the change in interaction patterns, thereby participating in the pathogenesis of FAD [40]. The p.L226R mutation of PSEN1, which is located at the transmembrane domain of the protein, was reported in an EOAD family characterized by language impairment at disease onset [58]. In a whole-exome sequencing study on 15 Chinese FAD patients, Jiang et al. identified six previously reported PSEN1 mutations in all subjects, including p.M139I, p.T147I, p.L173W, p.F177S, p.R269H, and p.R157S [39]. The probands carrying these mutations are likely to show highly heterogeneous phenotypes, based on the descriptions of the authors [39]. A recent study from CFAN reported ten missense mutations in PSEN1, and four of all ten mutations (including p.M139L, p.G111V, p.K311R and p.V97L) have been demonstrated to affect Aβ levels in previous reports [45].

#### 2.1.3. PSEN2

**Figure 3:**
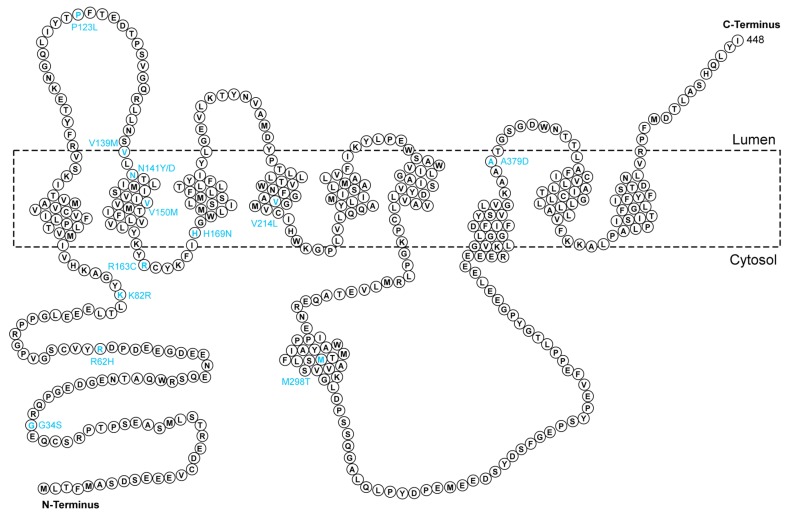
Amino acid sequence of presenilin 2 (PSEN2). The reported distribution of mutations in the Chinese population is labeled in cyan at the corresponding sites.

*PSEN2*, located on chromosome 1q31-q42, is highly homologous to *PSEN1* [67]. These two proteins have very similar structures and functions in the brain. PSEN2 and PSEN1 are the core components of γ-secretase. The reported *PSEN2* gene mutations (Figure 3) are much less common than those of *PSEN1*, with fewer than 40 mutations in *PSEN2* identified to date. Niu et al. first reported a PSEN2 mutation (p.N141Y) (Table 1) associated with autosomal dominant-EOAD in a Chinese Han family who was diagnosed with EOAD [65]. Another study on both AD and frontotemporal dementia (FTD) patients identified three novel mutations in PSEN2 [63]. The p.H169N mutation was found both in AD and FTD patients, while p.V214L and p.K82R mutations were only observed in EOAD patients [63]. The patients also depicted different Pittsburgh compound B (PIB) imaging variations as only AD patients showed PIB retention [63]. Xia et al. reported a new mutation in PSEN2 (p.P123L) in a Chinese familial EOAD patient. The patient showed symptoms of cognitive decline and memory impairment, as well as atypical neurological symptoms [64]. The p.H169N mutation of PSEN2 reported by Ma et al. has also been detected in both AD and FTD patients, in support of the previous finding by Shi et al. [58,63]. A known PSEN2 mutation (p.V139M) which was first reported in an Italian LOAD patient, was observed in a Chinese patient with early-onset disease phenotypes [39,73]. Gao et al. found two novel variants (p.V150M and p.R163C) in PSEN2 in Chinese familial AD patients. The authors claimed that individuals carrying mutations in PSEN2 might have a relatively later disease onset compared with those with PSEN1 mutations [40]. Two novel mutations (p.N141D and p.A379D) were found to be pathogenic genetic risk factors for EOAD [66]. Jia et al. reported four previously identified mutations in PSEN2 in the CFAN study, including p.G34S, p.R62H, p.V214L, and p.M298T. In the study, they also found a synonymous variant p.S236S, which was possibly related to AD [74].

### 2.2. LOAD Susceptibility Genes

LOAD is much more complicated than EOAD due to the complex interplay between multiple risk genes and environmental factors. Most LOAD cases are sporadic without apparent family history. The *APOE* ε4 allele has been recognized as the only LOAD risk gene for a long time. More risk genes for LOAD have been reported with the application of GWAS technology [75]. It is worth mentioning that almost all risk genes identified in recent years have different degrees of correlation with the Aβ cascade or tau pathology [75]. We briefly introduce the newly discovered risk genes in the Han Chinese population and related pathological mechanisms to LOAD biology (Table 2).

#### 2.2.1. APOE Gene

The *APOE* gene is approximately 3.6 kb in length, located on chromosome 19q13.2, with three common alleles: ε2, ε3, and ε4 [88]. The *APOE* ε2 variant is implicated with decreased LOAD susceptibility, whereas the effect of ε4 is completely opposite to that of the common ε3 allele, mainly because of the different regulations of these variants on APP transcription and Aβ secretion [89]. Approximately 50% of all AD cases carry at least one *APOE* ε4 allele, making it one of the greatest risk factors for LOAD [90,91,92,93,94,95]. Individuals carrying one ε4 allele are 3–4 times more likely to be diagnosed with AD than non-carriers, and the risk of developing AD increases by 12–15 times for those with two ε4 alleles [96,97]. Increasing evidence indicates that genetic polymorphisms of *APOE* ε4 are closely related to multiple types of dementia, such as Lewy body dementia (LBD) [98,99] and Parkinson disease dementia [100,101], suggesting a possible overlap in the mechanisms of different types of dementia in relation to *APOE* gene polymorphism. Although APOE is widely involved in the regulation of cholesterol metabolism, lipid transport, and APP production/metabolism [89,102,103], the best-established relationships are *APOE* variants and Aβ formation [104], accumulation [105] and plaque deposition in the brain [106,107]. Wei et al. found that *APOE* ε4 carriers have higher plasma Aβ42 levels and lower soluble low-density lipoprotein (LDL) receptor-related protein 1 (sLRP1) levels compared with non-*APOE* ε4 individuals [108]. LRP is a receptor of various ligands, including APOE and Aβ. Kang et al. demonstrated that LRP is closely involved in regulating the elimination of Aβ in the brain and strongly correlated with the susceptibility of AD [109]. Their data showed that reduced LRP expression in AD patients impedes the clearance of multiple Aβ species, thereby increasing the content of Aβ in the brain and decreasing the plasma Aβ levels [109]. However, the *APOE* ε4 allele appears to increase the plasma Aβ content, probably due to an elevation of total Aβ burden caused by APOE [109]. Extensive research has been performed on the correlation between the *APOE* genotype and the age of onset of LOAD in Caucasians; however, there have been relatively fewer research reports from China. *APOE* ε4 allele polymorphisms have been previously reported to affect the risk of developing LOAD in elderly Chinese individuals in Hong Kong and Taiwan, although the frequency of the *APOE* ε4 allele among the general aged population is lower than that of western countries [76,77].

#### 2.2.2. TREM2

The triggering receptor expressed on the myeloid cells 2 (*TREM2*) gene, located on chromosome 6q21.1, encodes a 230-amino acid, type I single- transmembrane protein called TREM2 [110]. Soluble TREM2 levels in the cerebrospinal fluid (CSF) are now considered a new marker for the onset and diagnosis of AD [111]. Several rare mutations have been reported to be strongly associated with LOAD in cohorts from Caucasian descendants over the past decades [112,113]. Some novel mutations have been reported in Chinese patients diagnosed with LOAD for the first time, but none of them showed a significant correlation to the risk of LOAD after Bonferroni correction [114,115], except a rare p.A139T variant, which has been reported to significantly alter the expression of TREM2 on the cell surface [79]. A rare TREM2 variant p.H157Y has been reported to be associated with LOAD susceptibility, according to a meta-analysis on 14,510 subjects by Jiang et al. [80]. The potential mechanism of the p.H157Y variant has been reported to be linked to APP proteolysis and lower TREM2-dependent phagocytosis by elevating the ectodomain shedding function [116]. The p.A130V variant of TREM2 has been found in LOAD patients in China and another novel mutation occurring adjacent to exon 2 of TREM2 has been reported in a Chinese Han consanguineous family but it is associated with EOAD [78,117]. Interestingly, Hou et al. discovered that mutations in the membrane spanning 4-domains A6A (*MS4A6A*) gene may affect the level of soluble TREM2 in the CSF [118].

#### 2.2.3. DAPK1

Although *APOE* ε4 has been confirmed to be by far the most important risk gene for LOAD in different populations, approximately half of the LOAD cases do not carry this allele according to the AlzGene database [119], which indicates that there are other potential genes contributing to the susceptibility of LOAD [120,121]. Chromosome 9 contains many regions related to AD with a number of linkage peaks by GWAS, making it the best candidate chromosome for studying the genetic risk of AD [122]. The death-associated protein kinase 1 (*DAPK1*) gene, located on 9q34.1, was first identified as a potential LOAD-contributing gene after analyzing its single nucleotide polymorphisms (SNPs) on chromosome 9 among a Caucasian population [123]. In this study, two SNPs, rs4878104 and rs4877365, were identified as candidate LOAD-contributing factors and were speculated to modulate susceptibility to LOAD by affecting neuron numbers in the hippocampus and/or by modulating the stress response to environmental stimuli. Since this discovery, DAPK1 has attracted extensive attention as a potential risk factor for LOAD.

DAPK1, a Ca^2+^/CaM-dependent serine/threonine protein kinase, plays a critical role in death signaling (proapoptotic, apoptotic and autophagic pathways) and the regulation of internal and external stress-induced cell damage, including ischemia and Aβ [124,125,126], which implies that it may have a role in various neuropathologies, such as AD, ischemia, and epilepsy. The critical role of DAPK1 in AD was confirmed at the cellular and animal levels [127,128], and it is currently believed that DAPK1 may be a key player in mediating the pathological process of dementia. The SNPs of *DAPK1* were first identified as one of the genetic factors that contributed to LOAD susceptibility in 2006. A Chinese research team validated these two gene variations (rs4877365 and rs4878104) with LOAD patients from the northern Han Chinese population in 2011, and the C allele of rs4878104 but not rs4877365 was recognized as a protective genetic factor of LOAD (odds ratio (OR): 0.75, p = 0.002), which provides the first evidence that DAPK1 allele-specific variants can influence the risk of developing LOAD in the Chinese population [82]. Although a much larger sample-based analysis showed a different conclusion from the previous one in that the rs4878104 variant is not significantly related to AD risk, it is significantly associated with AD susceptibility in the subgroup analysis [81].

#### 2.2.4. Other Genes Implicated in LOAD

The Unc-5 Netrin receptor C (*UNC5C*) gene is highly expressed in adult hippocampal neurons [129]. Four novel rare variants (p.Q860H, p.T837K, p.S843G, and p.V836V) near rs137875858 were found to be genetic risk factors for LOAD in the Han Chinese population [78]. Common variants of the GTP cyclohydrolase 1 (*GCH1*) gene (rs72713460) and the Potassium inwardly rectifying channel subfamily J member 15 (*KCNJ15*) gene (rs928771) were first identified as risk factors for AD in the Chinese population since they may affect immune-related pathways, leading to the altered onset age of AD [85]. The Coiled Coil-Helix-Coiled-Coil-Helix domain containing 10 (*CHCHD10*) gene was reported to be a risk gene for FTD and amyotrophic lateral sclerosis in Europe [130], two novel mutations (63C > T, no amino acid change; 71G > A, p.P24L) were also found in Chinese FTD patients [86]. The p.A35D variant of CHCHD10 was reported in a patient with sporadic LOAD in China [87].

## 3. Challenges and Future Insights

Although a plethora of gene variations have been identified in AD, it is still very difficult to pinpoint the exact pathogenesis of AD, particularly the sporadic type, because of the complex interaction between genetic and environmental factors. The linkage analysis and candidate gene studies are obviously unable to effectively clarify the LOAD genetic mechanisms due to limitations such as small sample size, vulnerability to locus heterogeneity, and false-positive results. The lack of knowledge on the etiology of AD hinders the development of effective treatments to slow down or to stop the progression of the disease.

Most of the research involved limited numbers of subjects for the genetic studies, which makes it necessary to establish large-scale, multicenter studies across the whole country to collect more data on the genetics of both EOAD and LOAD in China. For the identification of novel rare variants related to AD, deep sequencing can be a very powerful tool, which will help to better understand the underlying biology and risk factors involved in AD. Besides, it is also of great value to compare the difference in genetic risk factors between Chinese population and other populations worldwide, as this may contribute to explain the heterogeneity in disease phenotypes and the development of specific treatments. For instance, the CFAN discovered a lower detection rate of *APP/PSEN* mutations in the Chinese FAD population than that reported in Caucasians [45]. Additionally, it is also evident from the published studies that not all the susceptibility genes commonly found in Caucasian populations are present in Chinese AD patients [45,85]. For example, the CFAN reported a novel mutation in *APP* (M722K) in Chinese FAD patients, which was not found in other populations. Several mutations in *PSEN1*, including N24S, V97L, G111V, M139L, L172W, and K311R, were also first identified in the Chinese population [45]. Variation in the susceptibility genes in LOAD has also been reported between Chinese and Caucasian populations. The *TREM2* mutations are strongly associated with the incidence of AD in Caucasian populations, while only one novel mutation (H157Y) has been proven to be likely pathogenic in Chinese AD patients [131]. The initiation of large-scale genetic analysis in China could aid to extend the knowledge of AD risk genes in different ethnic groups.

## 4. Conclusions

AD is a neurodegenerative disease with complex pathological features, which are greatly affected by genetic factors. It has become one of the major public problems worldwide, considering the global trend of aging. Moreover, the shortage of effective therapeutics will definitely cause more stress on caregivers as well as the society as a whole. China has the largest AD population in the world. During rapid economic development, the problem of population aging is becoming increasingly concerning. It is of great strategic significance to study the risk genes of AD to obtain a deeper understanding of the underlying mechanisms of disease development and to provide a strong theoretical basis for the development of anti-AD drugs. The linkage study was once a powerful method for the genetic mapping of complex traits with familial aggregation, through which *APP*, *PSEN1*, and *PSEN2* were identified as risk genes for FAD [132]. With the rapid advancement of technologies and molecular genetic science, GWAS is widely used for investigating the common genetic architecture of complex trait diseases by identifying more than 20 loci in AD around the world. In addition, the development of whole-genome sequencing (WGS) provides further support to identify rare mutations that GWAS is unable to discover, thereby overcoming the common bias brought by GWAS in genetic studies [133]. Rare mutations may account for a significant proportion of the etiology of complex diseases. The identification of AD risk genes is mostly based on Caucasian populations, and the correlation between rare mutations and disease phenotype may not be directly applied to Chinese people. There have been some rare genetic mutations unique to Chinese people, such as *KCNJ15* and *GCH1*, providing an excellent basis for individualized and precise treatments of AD. In conclusion, to better understand the pathogenesis of AD, as well as to develop diagnostic and therapeutic strategies, a nationwide network should be established, and systematic gene analysis should be performed, which is now lacking in China.

## Figures and Tables

**Table 1:** Mutations in *APP*, *PSEN1*, and *PSEN2* as reported in Chinese population.

Gene	Location (Exon)	Mutation(s)	Pathogenicity	References
*APP*	6	D244G	Uncertain	[39]
	7	T297M	Uncertain	[39]
		D322G	Uncertain	[39]
	16	D678H	Increasing Aβ42/Aβ40 ratio; promoting Aβ aggregation	[44,46]
		K687Q	Uncertain	[39]
	17	V695M	Uncertain	[40]
		V710G	Uncertain	[38]
		V717I	Increasing Aβ42 generation	[42,47]
		I718L	Uncertain	[38]
		L720S	Uncertain	[38]
		M722K	Increasing Aβ42/Aβ40 ratio and tau phosphorylation	[37]
		K724M	Increasing Aβ42/Aβ40 ratio	[41]
*PSEN1*	4	V97L	Elevating Aβ42 generation	[48,49]
		V103G	Uncertain	[40]
		F105C	Uncertain	[42]
		G111V	Elevating Aβ42/Aβ40 ratio by reducing Aβ40 generation.	[45,50]
	5	A136G	Moderately decreasing Aβ; causing dendritic spine loss	[51,52]
		M139L	Increasing Aβ42/Aβ40 ratio	[40,53]
		M139I	Increasing Aβ42/Aβ40 ratio	[39,54]
		T147I	Uncertain	[39]
		R157S	Uncertain	[39]
	6	I167del	Decreasing Aβ42 production in vitro	[42]
		S169del	Decreasing Aβ42 production in vitro	[55]
		L173W	Increasing Aβ42/Aβ40 ratio	[33,39]
		F177S	Decreasing Aβ42 in CSF	[39,56]
		F177V	Uncertain	[40]
	7	G206V	Uncertain	[57]
		H214R	Uncertain	[57]
		L226R	Uncertain	[58]
		M233L	Increasing Aβ42/Aβ40 ratio	[55,59]
		L248P	Uncertain	[42]
		Y256N	Uncertain	[57]
	8	R269H	Uncertain	[39]
	9	K311R	Increasing Aβ42/Aβ40 ratio and phosphorylated tau	[45,60]
	10	R352C	Slightly decreasing Aβ generation	[61]
	11	V391G	Uncertain	[62]
	12	A434T	Uncertain	[42]
*PSEN2*	3	G34S	Uncertain	[45]
	4	R62H	No effect on Aβ42/Aβ40 ratio	[45]
		K82R	Uncertain	[63]
	5	P123L	Uncertain	[64]
		V139M	Uncertain	[39]
		N141Y	Uncertain	[65]
		N141D	Uncertain	[66]
		V150M	Uncertain	[40]
		R163C	Uncertain	[40]
	6	H169N	Uncertain	[63]
	7	V214L	Uncertain	[63]
		S236S	Uncertain	[45]
		M298T	Uncertain	[45]
	11	A379D	Uncertain	[66]

Abbreviations: APP: amyloid precursor protein; PSEN1: presenilin 1; PSEN2: presenilin 2; CSF: cerebrospinal fluid.

**Table 2:** Common and rare variants associated with sporadic/late onset (LOAD), as reported in the Chinese population.

Gene	Chromosome	Mutation(s)/SNPs	Pathogenicity	References
*APOE*	19q13.2	*APOE* ε4	Strongly promoting the deposition of Aβ	[76,77]
*TREM2*	6p21.1	A130V	Uncertain	[78]
		A139T	Uncertain	[79]
		H157Y	Increasing the shedding of soluble TREM2 and decreasing the phagocytosis	[80]
*DAPK1*	9q34.1	rs4878104	Associated with increased total Aβ levels	[81,82,83]
*UNC5C*	4q22.3	rs137875858	Increasing cell death	[78,84]
*GCH1*	14q22.2	rs72713460	Uncertain	[85]
*KCNJ15*	21q22.13	rs928771	Uncertain	[85]
*CHCHD10*	22q11.23	63 C > T (no AA change)	Uncertain	[86]
		P24L	Uncertain	[86]
		A35D	Uncertain	[87]

Abbreviations: APOE: apolipoprotein E; TREM2: triggering receptor expressed on the myeloid cells 2; DAPK1: death-associated protein kinase 1; UNC5C: Unc-5 Netrin receptor C; GCH1: GTP cyclohydrolase 1; KCNJ15: Potassium inwardly rectifying channel subfamily J member 15; CHCHD10: Coiled Coil-Helix-Coiled-Coil-Helix domain containing 10; AA: amino acid.
